# Renal Tubular Epithelial Cells as Central Hubs of Kidney Disease

**DOI:** 10.3390/diagnostics16111603

**Published:** 2026-05-24

**Authors:** Charlotte Delrue, Matthijs Oyaert, Eric Hoste, Joris R. Delanghe, Marijn M. Speeckaert

**Affiliations:** 1 Department of Nephrology, Ghent University Hospital, 9000 Ghent, Belgium; charlotte.delrue@ugent.be; 2 Department of Laboratory Medicine, Ghent University Hospital, 9000 Ghent, Belgium; 3 Department of Intensive Care Medicine, Ghent University Hospital, 9000 Ghent, Belgium; 4 Research Foundation Flanders, 1000 Brussels, Belgium; 5 Department of Internal Medicine and Pediatrics, Ghent University Hospital, 9000 Ghent, Belgium; 6 Department of Diagnostic Sciences, Ghent University, 9000 Ghent, Belgium; joris.delanghe@ugent.be

**Keywords:** renal tubular epithelial cells, multi-omics diagnostics, extracellular vesicles, acute and chronic kidney injury

## Abstract

Renal tubular epithelial cells (RTECs) are increasingly recognized as key players in kidney diseases. They integrate metabolic, inflammatory, and fibrotic signals. This article reviews new data suggesting that RTECs could function as central integrators within diagnostic networks, linking cellular stress responses to detectable blood and urine biomarkers. We discuss the latest advances in multi-omics, extracellular vesicles, and single-cell technologies that enable precise identification of RTEC states. Finally, we discuss the potential of RTEC-centric diagnostics and highlight current limitations in early disease recognition, stratification, and the development of personalized therapeutic interventions.

## 1. Introduction

Acute kidney injury (AKI) and chronic kidney disease (CKD) are responsible for hundreds of millions of cases worldwide and have a significant part in causing illness and death, resulting in high healthcare expenditures. Highly sensitive and specific biomarkers to detect the earliest injury, track disease progression, or predict treatment response remain lacking [1]. While they were once considered to be only harmed by the disease, renal tubular epithelial cells (RTECs) coordinate processes that drive disease initiation and progression through their roles in metabolism, inflammation, immunology, and fibrosis. The tubular epithelium has been identified as a sensing and integrating cell type in response to environmental factors, including ischemia, toxins, metabolic stress, immune activation, and hemodynamic changes. For instance, RTEC senescence has been recognized as a major factor that worsens AKI and slows regeneration by inducing cell-cycle arrest and a senescence-associated secretory phenotype (SASP) rich in inflammatory cytokines and chemokines [2].

Moreover, the genetic basis of kidney disease increasingly points to the tubular compartment. A groundbreaking analysis of regulatory variants showed that dysfunction of tubular epithelial cells accounts for a major share of the heritable variation in kidney function observed in genome-wide association studies (GWAS). These findings provide strong evidence that RTECs are the “genetic epicenter” of kidney function and disease susceptibility. At cellular and immunologic levels, RTECs serve as potent antigen-independent stimulators of resident kidney T-cells, thereby regulating tissue-resident immunity without relying on classical antigen-presentation pathways. This underscores the broad role of tubular cells in shaping both intrinsic epithelial responses and extrinsic immune microenvironments during kidney injury [3,4].

**Figure 1:**
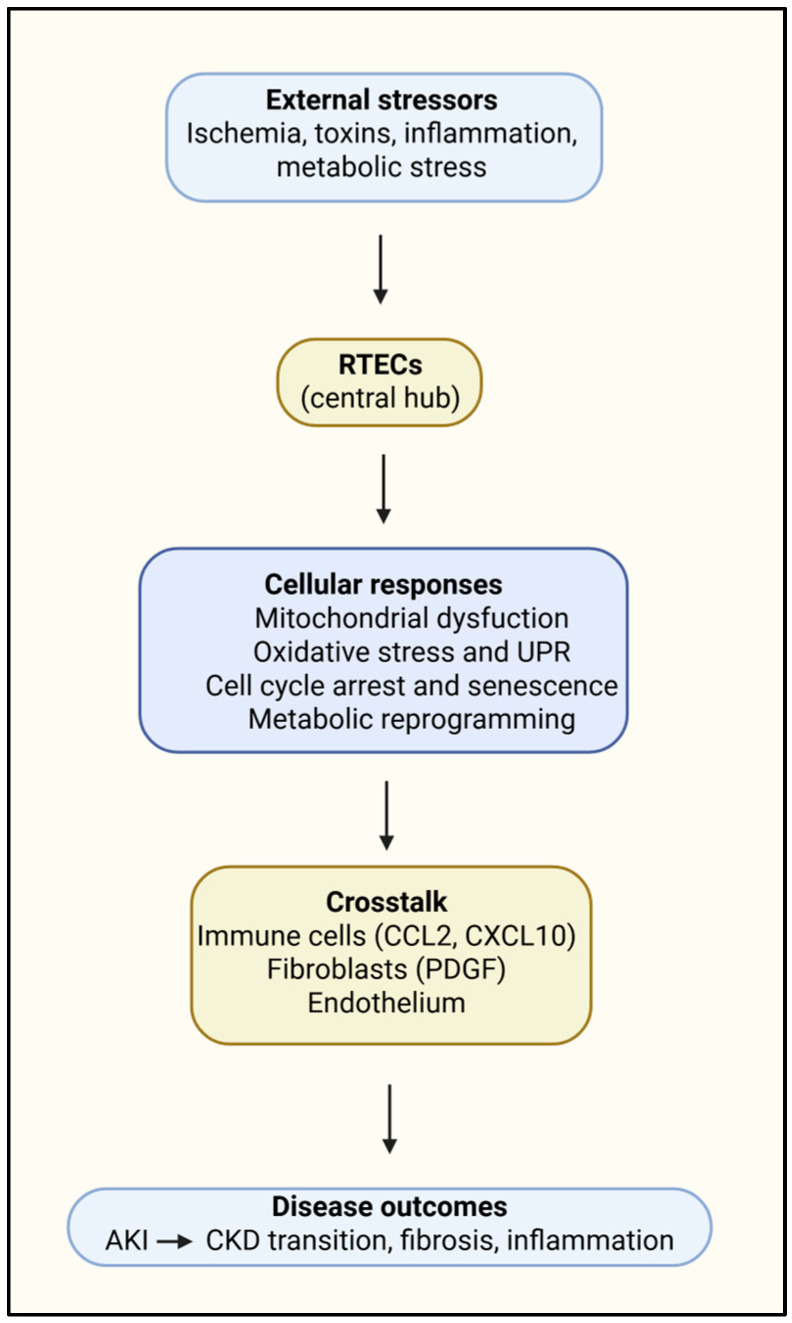
Conceptual model of RTEC-centered kidney injury pathways. External stressors such as ischemia, toxins, inflammation, and metabolic stress are the primary drivers of renal tubular epithelial cell (RTEC) injury, the main site of tubular injury in the kidney. After these cells are damaged by these agents, they mount significant cellular stress responses, including mitochondrial dysfunction, oxidative stress, activation of the unfolded protein response (UPR), cell-cycle arrest and senescence, and metabolic reprogramming. Subsequently, these altered epithelial cells communicate with immune cells (e.g., via C-C Motif Chemokine Ligand 2 (CCL2) and C-X-C Motif Chemokine Ligand 10 (CXCL10)), fibroblasts (e.g., via platelet-derived growth factor (PDGF) signaling), and endothelial cells, thereby further amplifying inflammation and fibrogenesis. The combined effect of these factors leads to clinical disease manifestations, including the development of chronic kidney disease (CKD) after an episode of acute kidney injury (AKI), interstitial fibrosis, and ongoing inflammation.

An important translational advance is the recognition of RTEC-derived biomarkers as powerful tools for monitoring tubular health. Recent clinical studies prove that quantifying urinary RTECs by flow cytometry provides a precise, reproducible method for assessing tubular damage and outperforms several commonly used biomarkers in selected settings. Extracellular vesicles (EVs) carry proteins, lipids, and nucleic acids that reflect their cellular origins. Multi-omics profiling has uncovered complex, highly conserved EV signatures, including thousands of molecular components that reveal cellular stress states and intercellular communication networks. Currently, multi-omics and single-vesicle technologies enable very high-resolution profiling of EV cargo, offering new insights into epithelial injury, immune activation, and fibrotic signaling pathways. Moreover, integrative multi-omics approaches are a powerful strategy for decoding EV heterogeneity, determining cell-type-specific contributions, and identifying candidate biomarkers and therapeutic targets [5,6,7,8]. RTECs handle sensing and integrating metabolic, inflammatory, and environmental insults (Figure 1). They communicate systemically by releasing EVs and soluble mediators and play a role in determining the course of kidney disease.

This review synthesizes current knowledge linking RTEC biology to kidney disease pathogenesis and diagnostics. We highlight mechanistic insights into RTEC injury, stress responses, immunologic interactions, and metabolic dysfunction; examine cutting-edge biomarker strategies spanning soluble markers, urinary cells, and EVs; and propose an integrated framework for RTEC-based diagnostics tailored to early detection, disease stratification, and personalized nephrology.

## 2. Methodology

We based this narrative review on a thorough literature search in the PubMed, Embase, and Web of Science databases. We considered publications from January 2010 to April 2026. We developed our search strategy by combining keywords such as ‘renal tubular epithelial cells’, ‘RTEC’, ‘kidney injury’, ‘multi-omics’, ‘extracellular vesicles’, ‘biomarkers’, ‘AKI’, and ‘CKD’. This paper also considered the reference lists of key papers as potential sources. The article types were original research, review articles, and clinical or translational studies, all recently published and focusing on the biology of RTECs, mechanisms of RTEC injury, diagnostic biomarkers, and omics technologies in nephrology. Only English-language studies were considered.

## 3. Biology of Renal Tubular Epithelial Cells

### 3.1. Structure and Function of RTECs

RTECs form the major epithelial lining of the nephron and are organized into highly specialized segments, each optimized for distinct transport, metabolic, and detoxification functions. This segmental organization enables precise regulation of solute homeostasis, acid-base balance, endocrine signaling, and xenobiotic handling. Each tubular segment, from the proximal tubule through the loop of Henle, distal convoluted tubule, and collecting duct, has distinct structural and molecular features reflecting its physiological role.

The proximal tubule is often called the “workhorse” of the nephron, due to its high reabsorptive capacity and metabolic activity. It reabsorbs nearly all filtered solutes, including glucose, amino acids, bicarbonate, phosphate, and small proteins, and secretes various naturally occurring metabolites, drugs, and toxins. Its extensive brush border, abundant mitochondria, and high surface-to-volume ratio reflect its substantial energy demand. The proximal tubule is also the nephron’s major site of injury in AKI and a central driver of maladaptive repair and fibrosis when regeneration fails, underscoring its vulnerability to filtered nephrotoxins [9]. Single-cell transcriptomic studies reveal substantial heterogeneity within proximal tubule subsegments (S1–S3), each defined by distinct molecular signatures and injury-response states. Injury can make it harder to distinguish cell types, leading to abnormal states in proximal tubule cells. It induces metabolic changes, inflammation, and impaired transport functions, as shown by the Kidney Precision Medicine Project (KPMP) atlas. In CKD, specific subsets of injured proximal tubule cells, such as PT_5, progressively expand and actively coordinate fibrotic remodeling through ligand-receptor interactions with macrophages, fibroblasts, and T cells, underscoring their central role in chronic disease progression [10,11].

The thick ascending limb reabsorbs sodium, potassium, and chloride via the sodium-potassium-chloride cotransporter 2 (NKCC2), contributing to countercurrent multiplication and urinary concentration. It also participates in calcium and magnesium handling [12,13,14].

The distal convoluted tubule fine-tunes electrolyte balance, particularly sodium, potassium, and calcium, via specialized transporters, including the sodium-chloride cotransporter (NCC) and transient receptor potential cation channel subfamily V member (TRPV5). It plays a crucial role in blood pressure regulation and acid-base balance [15].

The collecting duct system provides hormonal regulation of sodium, potassium, and water balance through aldosterone-sensitive principal cells and vasopressin-sensitive aquaporin-expressing cells. Intercalated cells mediate acid-base regulation [16,17].

To illustrate the physiological and pathophysiological importance of RTECs in human health and disease, we describe two clinical examples with significant impact. One effect of SGLT2 inhibitors (SGLT2i) is that they can protect the kidneys beyond simply causing glycosuria. These drugs primarily affect proximal tubular epithelial cells by lowering intracellular glucose levels, thereby reducing glycolysis and mitochondrial reactive oxygen species (ROS) production, while simultaneously activating energy-sensing mechanisms, including the 5′-adenosine monophosphate-activated protein kinase (AMPK) and mechanistic target of rapamycin complex 1 (mTORC1) signaling pathways [18,19,20,21]. These changes trigger a metabolic state that resembles fasting, recondition mitochondria, and reduce inflammatory and fibrotic signaling in tubular cells, including epithelial–mesenchymal transition (EMT) and tubulointerstitial fibrosis [22,23,24]. Second, proton pump inhibitors (PPIs) exemplify how commonly prescribed drugs can induce kidney injury through primary effects on RTECs. Acute interstitial nephritis caused by PPIs is an immune reaction, and one of its initial steps is damage to tubular epithelial cells, which leads to inflammation via antigen presentation. Afterward, T cells and other immune cells are recruited and activated in the interstitium [25,26,27]. Additionally, injured RTECs contribute significantly to this process by secreting proinflammatory cytokines and danger signals, thereby increasing tubulitis and interstitial inflammation. If the PPI is not eliminated, ongoing epithelial damage and immune activation will lead to tubular atrophy, interstitial fibrosis, and the development of CKD [26,27,28]. These cases demonstrate that RTECs are not merely targets of injury but key regulators of metabolic homeostasis, immune signaling, and disease progression.

### 3.2. RTEC Stress Responses

**Figure 2:**
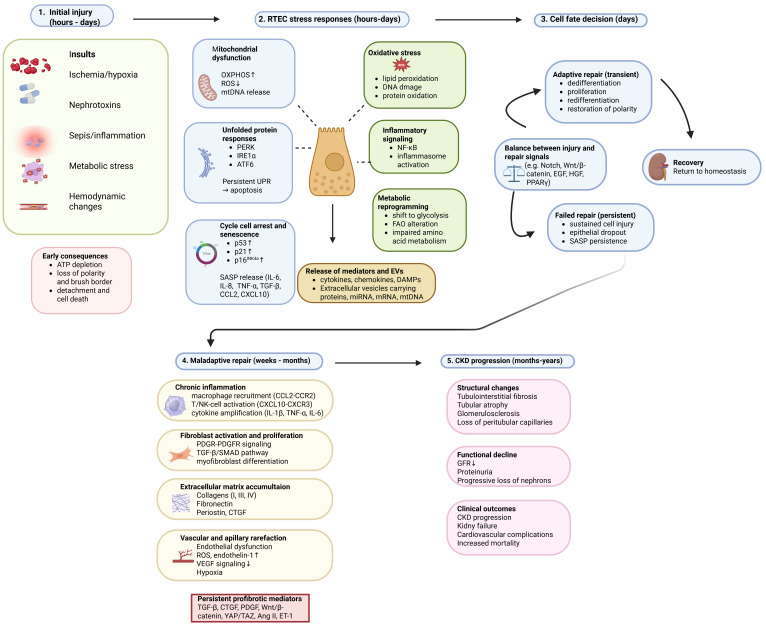
Molecular pathways underlying the transition from acute kidney injury (AKI) to chronic kidney disease (CKD). This scheme illustrates the pathways at both the cellular and molecular levels that mediate the progression of AKI to CKD, with renal tubular epithelial cells (RTECs) playing a key role. Some of the initial risk factors for injury to RTECs include ischemia/hypoxia, nephrotoxins, sepsis, and metabolic or hemodynamic factors, and the duration of these acute factors can range from hours to days (therefore, influencing the cellular pathways listed). As a result of the injury, some of the earliest cellular processes include ATP depletion, changes in RTEC polarity, and RTEC cell death. RTECs respond to damage through the activation of various stress-related pathways (e.g., mitochondrial dysfunction due to reduced oxidative phosphorylation and increased levels of reactive oxygen species (ROS)), activation of the unfolded protein response (UPR; via the major three pathways: PERK, IRE1, and ATF6) and NF-κB-mediated inflammatory signal pathways from activated inflammasomes, cell cycle arrest and senescence (via p53, p21, and p16) and producing senescence-associated secretory phenotype (SASP) molecules; and modifying metabolic pathways (by decreasing fatty acid oxidation and increasing glycolysis). Injury and repair molecules (e.g., Notch, Wnt/-catenin, EGF, HGF, PPAR) determine cell fate decisions over the course of days. Upon the release of mediators (including cytokines, chemokines, damage-associated molecular patterns (DAMPs), and extracellular vesicles) and through adaptive repair, epithelial integrity can be restored via dedifferentiation, proliferation, and redifferentiation, leading to the generation of functionally recovered tissue. In CKD, maladaptive tissue repair processes create pathways that sustain inflammation, drawing inflammatory immune cells and further damaging signals to the kidney. This causes excessive buildup of extracellular matrix (ECM) and encourages fibroblasts (connective tissue cells responsible for forming and repairing new fibers when a wound occurs) to transform into myofibroblasts (cells that contract and produce large amounts of collagen fibers). The end result of these activities will cause the blood vessels in the kidney to become rarefied (decreased number and total vascular area) due to endothelial cell impairment and insufficient vascular endothelial growth factor (VEGF) activity. Furthermore, among other pro-fibrotic/cytokine mediators (transforming growth factor beta, connective tissue growth factor, platelet-derived growth factor, Wnt/beta-catenin, YAP/TAZ, angiotensin II, and endothelin 1), these factors continue to influence chronic renal failure development for weeks to months, resulting in two major complications: increased risk of dying and an increased risk of developing kidney failure.

When faced with ischemic, toxic, inflammatory, or metabolic injuries, RTECs initiate a series of stress responses that guide epithelial healing or drive a maladaptive transition to fibrogenic states. These responses include mitochondrial dysfunction, oxidative and proteotoxic stress, and cell-cycle arrest programs that strongly influence the progression of kidney disease (Figure 2) [18].

#### 3.2.1. Mitochondrial Dysfunction

Tubular injury is accompanied by changes in mitochondrial metabolism, which have been identified as significant contributors to acute and chronic epithelial dysfunction. Normal tubular epithelial cells primarily generate energy through fatty acid oxidation rather than glycolysis, given their very high energy requirements. This unique metabolic feature makes them highly dependent on properly functioning mitochondria and, at the same time, highly sensitive to any decrease in oxygen or changes in the types of nutrients they can use. In sepsis-associated AKI, tubular cells not only reduce fatty acid metabolism and the energy production associated with it but also attempt to compensate by increasing glycolysis. However, this compensatory metabolic shift is harmful because continuous reliance on glycolysis leads to tubular cell death and fibrosis, resulting in kidney damage over time [18,29,30,31].

On a molecular scale, a series of changes in cellular metabolism is controlled by a single network of dysregulation of major transcription regulators such as peroxisome proliferator-activated receptor alpha (PPARα), peroxisome proliferator-activated receptor gamma co-activator 1 (PGC-1), and AMPK, which, in the absence of a metabolic shift, promote mitochondrial biogenesis and fatty acid oxidation. Reduction in PGC-1 levels has been identified as a major factor in tubular metabolic collapse, leading to failure of mitochondrial replication, decreased oxidative capacity, and reduced ATP content. On the other hand, hypoxia-inducible factor 1 (HIF-1) and glycolytic enzymes (e.g., hexokinase 2 (HK2), 6-phosphofructo-2-kinase/fructose-2,6-bisphosphatase 3 (PFKFB3)) induce a glycolytic phenotype associated with dedifferentiation and fibrotic signaling [18,30,32,33]. New findings also pinpoint defective mitophagy as a key determinant of tubular cell fate. Failure to eliminate damaged mitochondria results in an accumulation of dysfunctional organelles, excessive production of ROS, and the release of mitochondrial DNA (mtDNA), a DAMP that can activate the cyclic GMP-AMP synthase (cGAS)—stimulator of interferon genes (STING) and inflammasome pathways. This mitochondrial-immune axis directly links metabolic failure, innate immune activation, and fibrosis [30,32,34,35,36].

In proximal tubule injury models, mitochondrial dysregulation contributes to the formation of abnormal cell states identified by single-cell RNA sequencing, including dedifferentiated, metabolically reprogrammed, and pro-fibrotic epithelial phenotypes that accumulate during CKD progression [6,34,37,38].

Alongside oxidative damage, adaptive stress responses in RTECs are also critical in determining the cell’s fate after nephrotoxic injury. One of the major protective mechanisms induced by autophagy activation in tubular epithelial cells is enhanced cellular survival under stress, achieved by removing damaged organelles and limiting ROS accumulation. Pharmacological activation of autophagy improves the viability of tubular epithelial cells, reduces oxidative stress, and increases resistance to apoptosis, ultimately reducing kidney injury [39].

#### 3.2.2. Oxidative Stress and the Unfolded Protein Response

By producing abundant ROS, damaged RTECs not only harm themselves but also amplify environmental stress. ROS attack surrounding lipids, proteins, and nucleic acids. These conditions exacerbate cellular stress and trigger maladaptive signaling pathways. ROS overproduction also activates nuclear and cytosolic stress sensors, including nuclear factor kappa-light-chain-enhancer of activated B cells (NF-κB), contributing to a robust proinflammatory phenotype characteristic of tubular injury [3,18,30,40]. Increased protein misfolding activates the UPR, a key adaptive mechanism regulated by ER stress sensors, including protein kinase R-like endoplasmic reticulum kinase (PERK), inositol-requiring enzyme 1 (IRE1), and activating transcription factor 6 (ATF6). If the UPR persists or is overactivated, it drives cells toward apoptosis or senescence rather than recovery. RTEC senescence is characterized not only by irreversible cell-cycle arrest but also by the secretion of a SASP rich in inflammatory cytokines and chemokines, which propagate interstitial inflammation and promote the AKI-to-CKD transition. RTEC senescence can be caused by several stressors: telomere damage, DNA damage, defective mitophagy, metabolic dysfunction, and ER stress, each of which exacerbates the injured kidney’s proinflammatory and anti-regenerative environment [30,35,40,41,42].

#### 3.2.3. Cell-Cycle Arrest and Maladaptive Repair Mechanisms

Cell-cycle arrest is a hallmark of the tubular response to injury. In the early stages of AKI, transient G2/M arrest allows RTECs to assess and repair damage, thereby preventing the propagation of mutations and promoting epithelial integrity. However, sustained or repeated injury drives persistent cell-cycle arrest, leading to maladaptive epithelial remodeling [43,44,45]. The creation of single-cell maps of human and rodent kidneys affected by injury has revealed that tubular cells undergo transformation, leading to the expression of stress markers (such as hepatitis A virus cellular receptor 1/kidney injury molecule-1 (HAVCR1/KIM1), vascular cell adhesion molecule 1 (VCAM1), complement component 3 (C3)) and the initiation of pro-fibrotic pathways. These injured epithelial cells, which have lost their original features, send chemical signals to the surrounding environment. They act together with macrophages, fibroblasts, and immune cells to expand the interstitial space, lay down the matrix, and produce fibrosis [6,10,11,46,47]. Moreover, maladaptive repair is exacerbated by metabolic inflexibility, mitochondrial damage, and chronic oxidative stress. These factors collectively place the epithelium in a state of chronic inflammation that, in addition to impairing tubular function, exacerbates the disease. Additionally, the convergence of senescence, maladaptive dedifferentiation, and persistent cell-cycle arrest forms a harmful axis that drives progression (Table 1) [17,18,29,30,43,45].

Senescence in proximal tubular epithelial cells is not only harmful but also stage-dependent in altering their function. Acute senescence, a temporary p53/p21-mediated cell-cycle arrest, might be a response of tubular epithelial cells to injury, preventing further DNA damage. If senescence is persistent or chronic, cells produce the SASP for long periods, fibroblasts in the interstitium become activated, and the extracellular matrix (ECM) is deposited. This ultimately accelerates the progression from acute kidney injury to chronic kidney disease [48].

### 3.3. RTEC Crosstalk with Other Kidney Cell Types

**Figure 3:**
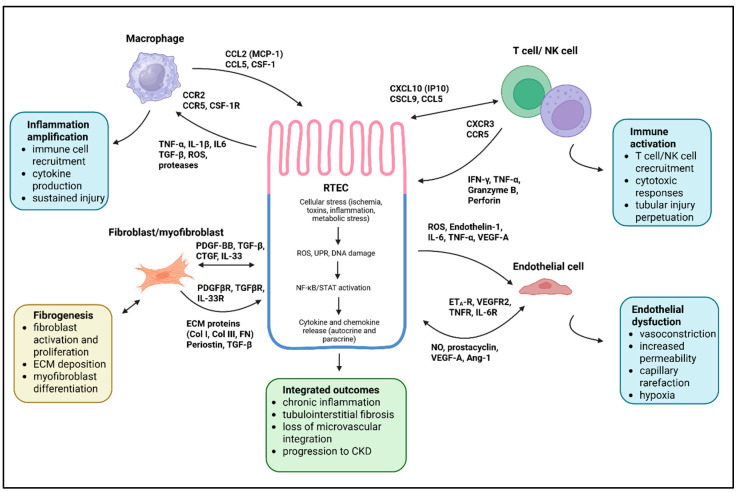
Crosstalk between renal tubular epithelial cells (RTECs) and cells in the kidney microenvironment. Renal tubular epithelial cells (RTECs) form the principal fabric of the kidney microenvironment. In addition to their crosstalk with immune cells, these cells can interact with fibroblasts and endothelial cells. When exposed to insults such as anoxia, toxins, inflammation, or metabolic stress, RTECs experience cellular stress characterized by increased reactive oxygen species (ROS) levels, activation of the unfolded protein response (UPR), DNA damage, and alterations in inflammatory signaling pathways (e.g., NF-κB and STAT). By producing cytokines, chemokines, and growth factors, stressed RTECs promote intercellular communication. Several chemokines are secreted by RTECs, including CCL2, CCL5, and CSF-1, which attract and activate macrophages via the CCR2/CCR5/CSF-1R pathways. Thereafter, these macrophages secrete increased levels of TNF-α, IL-1, IL-6, TGF-β, ROS, and proteases, thereby worsening inflammation. Similarly, RTECs produce CXCL9 and CXCL10, which recruit T cells and NK cells via CXCR3/CCR5 signaling, resulting in cytotoxic effects and sustained tubular damage. In addition to communicating with fibroblasts via paracrine signaling, RTECs can produce pro-fibrotic mediators, including PDGF-BB, TGF-β, connective tissue growth factor (CTGF), and IL-33, which drive fibroblast activation, proliferation, and myofibroblast conversion. Ultimately, all of these result in extracellular matrix (ECM) deposition. Simultaneously, several RTEC-derived substances, such as ROS, endothelin-1, IL-6, TNF-α, and VEGF-A, are also involved in the pathogenesis of endothelial dysfunction, a condition marked by vasoconstriction, increased permeability, and capillary loss. These interlocking signaling mechanisms will culminate in highly intricate pathological manifestations, including persistent inflammation, interstitial fibrosis, loss of microvessels, and the development of CKD.

RTECs, especially proximal tubular epithelial cells (PTECs), are not merely metabolic and transport units. They also serve as immunomodulatory and signaling centers within the kidney microenvironment. Following injury, whether acute (AKI) or chronic (CKD), RTECs alter their gene expression, metabolism, and structure, enabling extensive communication with immune, stromal, endothelial, and interstitial cell types. This crosstalk among cell types is crucial for the kidney to either adaptively repair itself or develop maladaptive fibrosis, thereby determining long-term renal outcomes. Single-cell transcriptomic and spatial multi-omic studies are gradually revealing the extent and depth of these cell-to-cell interactions, positioning RTECs as the primary regulators of intrarenal inflammation, interstitial expansion, and fibrotic progression (Figure 3).

#### 3.3.1. Interactions with Immune Cells

Injured RTECs release a broad spectrum of chemokines, cytokines, stress ligands, and danger-associated molecular patterns (DAMPs) that recruit and activate immune cells. In addition to chemokine-dependent recruitment of immune cells, RTECs themselves are immune cells that coordinate both immediate and later immune responses. Cells can identify many patterns through pattern recognition receptors (PRRs), such as Toll-like receptors (TLR2, TLR4) and nucleotide-binding and oligomerization domain (NOD)-like receptors. These receptors are assigned to directly detect pathogen-associated and DAMPs. When such receptors engage their ligands, they initiate intracellular signaling cascades through the NF-κB and interferon pathways, leading to increased expression and secretion of inflammatory cytokines, including interleukin (IL)-6, tumor necrosis factor (TNF)-α, and type I interferons [3,47]. Furthermore, RTECs can influence T-cells not only by presenting antigens. It has been demonstrated that kidney tubular cells sustain tissue-resident memory T cells through co-stimulatory signals and cytokine support, thereby maintaining chronic inflammation even in the absence of classical antigen presentation. In this way, the role of RTECs shifts from mere passive targets to immunological centers that shape the kidney immune milieu [4].

Single-cell RNA sequencing of CKD models has identified distinct injury-associated tubular subsets that upregulate a classical injury program. The CCL2-CCR2 axis exemplifies this phenomenon. Injured PTECs release CCL2, a monocyte-attracting chemokine that preferentially targets CCR2-expressing monocytes and macrophages. Damaged epithelial cells also interact via IL-34-colony-stimulating factor 1 receptor (CSF1R) signaling, which not only supports macrophage survival but also promotes their local proliferation, thereby exacerbating chronic inflammation in CKD [11,49]. In addition to myeloid cells, tubular cells communicate with T and natural killer (NK) cells via the CXCL10-CXCR3 pathway. Injury-related epithelial cells release CXCL10, which helps attract and retain CXCR3-expressing immune cells, leading to both cytotoxic activity and chronic inflammation. High-definition human kidney cell maps also confirm that zones of epithelial injury are not only physically close to infiltrating immune cells but also exhibit stronger immune-epithelial interactions [47,50].

#### 3.3.2. Crosstalk with Fibroblasts and Endothelial Cells

Fibroblasts are the primary effector cells in kidney fibrosis, and injured RTECs directly activate fibroblast populations through specific ligand-receptor circuits. The PDGF-PDGFR axis, expressed by PT_5 epithelial cells and PDGFR^+^ fibroblasts, constitutes a principal epithelial-to-stromal growth and activation signal that drives fibroblast proliferation, ECM deposition, and myofibroblast differentiation. Fibroblast subsets such as Myofibroblasts_Timp1 expand markedly in CKD and are tightly associated with epithelial injury signals emanating from maladaptive RTEC populations [47].

Endothelial cells are also key participants in the injury niche. Multimodal spatial mapping of human kidney tissue reveals that RTEC injury neighborhoods exhibit profound epithelial-endothelial-immune interactions, coupled with microvascular destabilization and capillary rarefaction, collectively impairing oxygen delivery, exacerbating metabolic stress, and potentiating fibrosis [51].

Additionally, studies of stromal heterogeneity show that stromal cells dynamically shift their identities in response to epithelial signals, with epithelial-derived metabolic and lipid stress signals influencing fibroblast and endothelial cell phenotypes during both the early injury and chronic phases of disease [7].

## 4. RTECs as Drivers of Kidney Disease

### 4.1. Polycystic Kidney Disease

The autosomal dominant form of polycystic kidney disease (ADPKD) is a classic example of a tubulocentric disease among the polycystic kidney diseases (PKDs). RTECs not only suffer passive damage but also actively contribute to all stages of disease, from cyst formation to cyst growth and progression. Many of the genetic changes responsible for the disease lie in the PKD1 or PKD2 genes. These genes encode polycystin-1 (PC1) and polycystin-2 (PC2), respectively. The polycystins, which are protein complexes, localize to the primary cilium and play roles in intracellular calcium signaling, mechanosensation, and the maintenance of epithelial cell structure. A deficiency in polycystin function leads to alterations in these cellular activities and to cellular reprogramming of tubular epithelial cells, ultimately resulting in cystic growth [52].

One of the main reasons epithelial cells spread uncontrollably in PKD is that loss of signaling through PCs causes RTECs to dedifferentiate and re-enter the cell cycle. As a result, their phenotype shifts to a proliferative state, leading to the formation of the first tubular epithelial outpouching and cystic structures. Such abnormal proliferation is supported by a network of signaling pathways, including activation of the RAS/RAF/MEK/ERK cascade, mTOR signaling, and the Wnt/β-catenin pathway. These pathways all contribute to increased cell division and cyst growth. Most importantly, this proliferative response is tightly linked to the expression of intracellular second messengers. In particular, cyclic AMP (cAMP) paradoxically stimulates cell proliferation in cystic epithelial cells while inhibiting cell proliferation in normal tubular epithelium [1,52,53].

One hallmark of PKD is the loss of epithelial polarity and structural organization. Under normal conditions, RTECs in nephrons exhibit apicobasal polarity, which is critical for vectorial transport and tubular integrity. In PKD, PC deficiency disrupts ciliary signaling and cytoskeletal organization, leading to mislocalization of membrane proteins, defective cell–cell and cell–matrix interactions, and loss of planar cell polarity. These changes result in abnormal cell-division orientation and structural disorganization, promoting radial expansion of tubular segments rather than longitudinal growth. In parallel, impaired ciliary mechanosensation prevents proper regulation of luminal diameter, further contributing to cyst initiation and expansion [54,55,56,57].

Beyond cell proliferation, the increase in cyst size primarily results from fluid secretion into the cyst cavity, driven by RTECs adopting a secretory phenotype. Epithelial cells lining the cysts increase chloride secretion across the epithelium, largely through cAMP-dependent activation of apical chloride channels, such as CFTR, and, in some cases, calcium-activated channels, such as TMEM16A. This chloride secretion creates an osmotic gradient that draws sodium and water into the cyst cavity, leading to stepwise fluid accumulation and cyst growth. These secretory processes are also supported by basolateral ion transporters and potassium channels, which help maintain the electrochemical gradient required for continuous secretion [58,59,60].

### 4.2. Lupus Nephritis

Lupus nephritis (LN) is a significant organ manifestation of systemic lupus erythematosus (SLE). Traditionally, it is characterized as immune complex-induced glomerular injury. However, the contribution of tubulointerstitial damage, particularly the role of RTECs, to disease progression has become a major focus and is often overlooked. Currently, glomerular lesions form the basis of histopathological classification systems. Tubulointerstitial inflammation and fibrosis are more strongly associated with renal outcomes, including progression to CKD and kidney failure [61].

In LN, immune complexes consisting of nucleic acids and autoantibodies are deposited not only in the glomerular structures but also along the tubular basement membranes and in the interstitium, thereby directly exposing RTECs to pathogenic stimuli. Moreover, DAMPs complement the activation products, and proinflammatory cytokines produced by the inflamed glomeruli diffuse into the tubulointerstitium, further activating tubular epithelial cells. This leads RTECs to undergo significant phenotypic and functional changes, turning them into immunologically active cells capable of influencing both innate and adaptive immune responses [62].

One of the breakthroughs highlighted in the review article by Hong et al. [62] is the recognition that RTECs in the kidney function as immune cells that are not typically considered part of the immune system. These features have been clearly described as those of professional antigen-presenting cells. For instance, when RTECs become active, they produce increased levels of MHC class I and II molecules, along with co-stimulatory molecules. As a result, these cells become capable of presenting antigens and can also contribute to interactions between RTECs and the T cells that have entered the kidney. In addition, they release a wide variety of cytokines (e.g., IL-6, TNF-α), chemokines (e.g., CCL2/MCP-1, CXCL10), and cell adhesion molecules that coordinate leukocyte recruitment to the kidney, retention, and activation.

Localized immune cell aggregates, such as T cells, B cells, dendritic cells, and macrophages, that resemble lymphoid structures ectopically are a hallmark of the tubulointerstitium in LN. They are considered responsible for antigen presentation and local autoimmunity, which can lead to tissue damage even in the absence of systemic disease flares. RTECs are important in maintaining these niches by delivering chemotactic and survival signals. IL-6 produced by RTECs, for example, not only initiates inflammatory responses but also induces fibrocyte and myofibroblast differentiation, thereby linking immune activation and fibrotic remodeling. RTECs play a key role in LN by contributing to tubulointerstitial fibrosis. Disrupted inflammatory signals, combined with metabolic stress and hypoxia, cause RTECs to undergo harmful repair processes. These cells change their nature and age, and release agents that promote further fibrosis, such as TGF-β. Although the debate over whether full EMT occurs in vivo persists, partial EMT and paracrine signaling from damaged RTECs have been established as important factors in activating fibroblasts and promoting extracellular matrix production. RTECs can promote fibrogenesis by secreting cytokines that induce the differentiation of circulating fibrocytes. These fibrocytes accumulate in the interstitium and increase matrix production. The result is interstitial fibrosis and tubular atrophy (IFTA), which is strongly linked to reduced glomerular filtration rate and poor patient outcomes. Recent research has revealed that inflammatory activity within fibrotic areas (iIFTA) is not just a passive scarring event but rather an active pathological process linked to disease progression. In LN biopsies, fibrosis is generally accompanied by infiltrating immune cells, particularly macrophages and T cells, and the extent of inflammation in fibrotic areas determines the rate of kidney function decline. This illustrates that RTECs located in fibrotic niches remain metabolically and immunologically active, thereby perpetuating damage even after structural changes have occurred [63,64,65,66,67].

From a mechanistic perspective, several intracellular signaling pathways in RTECs are altered in LN, including NF-κB, JAK/STAT, and interferon signaling cascades. Type I interferons, the hallmark of SLE, profoundly affect RTECs and upregulate interferon-stimulated genes, which, among other effects, increase antigen presentation and the production and recruitment of cytokines and immune cells. In addition, activation of Toll-like receptors (TLRs) on RTECs by nucleic acid-containing immune complexes plays a fundamental role in the pathogenesis of lupus nephritis, greatly increases inflammatory signaling, and, as a result, links systemic autoimmunity with local renal responses. In fact, crosstalk between RTECs and immune cells, including macrophages, T cells, and dendritic cells, results in a complex network of bidirectional interactions that not only drive disease progression but also sustain chronic inflammation [68,69,70,71].

Aside from their roles in pathology, RTECs could be key to discovering disease biomarkers and novel treatments for LN. Urinary biomarkers derived from damaged RTECs or their response, such as chemokines and tubular proteins, can be used to track disease progression and predict outcomes noninvasively. Treatment modalities that modify RTEC actions, such as blocking proinflammatory signaling pathways, preventing maladaptive repair, and targeting tubulointerstitial fibrosis, are increasingly recognized as adjuncts to classic immunosuppressive therapy [61,72,73,74,75].

### 4.3. IgA Nephropathy

RTECs have been identified as major contributors to IgA nephropathy (IgAN), not only reflecting the damage but also actively mediating tubulointerstitial injury and disease progression. Even though the deposition of galactose-deficient IgA1 (Gd-IgA1) immune complexes in glomeruli results in mesangial inflammation, the degree of tubulointerstitial damage, which is mainly the work of RTECs, is related to the long-term renal prognosis [76,77,78].

Mesangial–tubular crosstalk plays a central role in RTEC activation. Polymeric IgA1 (pIgA1) complexes attached to mesangial cells are capable of triggering the release of cytokines (e.g., IL-6, TNF-α), complement fragments, angiotensin II (Ang II), and aldosterone. These soluble mediators can cross the glomerular-tubular barrier and activate RTECs that express mineralocorticoid (MR) and angiotensin II type 1 (ATR) and type 2 (ATR) receptors. Exposure to mesangial-conditioned media from pIgA1-stimulated cells markedly increases RTEC proliferation and the production of proinflammatory mediators (including IL-6, TNF-α, ICAM-1, and Ang II), in contrast to direct exposure to IgA, which has little effect on RTECs [76,77,78].

In addition, aldosterone and Ang II together markedly increase oxidative stress and cell death in RTECs via the NADPH oxidase, ROS, and caspase-3 pathways. Also, aldosterone induces autocrine Ang II signaling and TGF-β production via MR activation, whereas MR antagonists or ATR blockers prevent these effects. As a result, a self-amplifying cycle of hormonal, oxidative, and inflammatory damage develops in the tubulointerstitium [76,78].

After cellular overload in RTECs, the cells undergo functional failure, with oxidative, ER stress, inflammatory, and fibrogenic pathways continuously activated, causing cell death, dedifferentiation, and changes resembling EMT. Such changes lead to matrix buildup and tubulointerstitial fibrosis, which are major factors of renal function loss in IgAN [76,77,78].

### 4.4. ANCA-Vasculitis

Antineutrophil cytoplasmic antibody (ANCA)-associated vasculitis (AAV) is a systemic autoimmune small-vessel vasculitis. The kidney is a major target organ. It is classically characterized by pauciimmune necrotizing and crescentic glomerulonephritis, driven by autoantibodies against myeloperoxidase (MPO) or proteinase 3 (PR3). Although the glomerular lesion is the initial and major feature, renal damage in AAV extends beyond the glomerulus and significantly involves the tubulointerstitial compartment. In this compartment, tubular necrosis, interstitial inflammation, and fibrosis are not only features but also major determinants of disease severity and kidney outcome. Disease progression and relapse risk correlate with the extent of tubulointerstitial injury, particularly tubular atrophy and interstitial fibrosis, underscoring the role of nonglomerular damage in disease progression [79].

RTECs are primarily responsible for disease progression. Following ANCA-induced neutrophil activation and vascular damage, numerous inflammatory cytokines and mediators, including TNF-α, IL-1, and IL-18, are released. These mediators diffuse into the tubulointerstitium, where they activate RTECs. In turn, these cells upregulate adhesion molecules such as ICAM-1 and VCAM-1, which attract and retain leukocytes in the interstitium. This leads to increased infiltration of immune cells and establishes a persistent inflammatory state. Activated epithelium is also indicated by elevated chemokine production, including CX3CL1 (fractalkine), which recruits monocytes and T cells to the kidney and is markedly elevated in MPO-AAV renal tissue. Fractalkine expression is predominantly observed in tubular epithelial cells, highlighting their role as more than bystanders and as a key component of the inflammatory milieu [80,81].

RTECs also undergo profound phenotypic and molecular alterations under inflammatory stress. Changes in cytoskeletal structure, including altered keratin expression, are indicative of epithelial stress and dedifferentiation in AAV, as in other chronic kidney diseases. These stress responses, together with intracellular pathways such as autophagy, oxidative stress signaling, and inflammatory cascades, contribute to epithelial dysfunction and maladaptive remodeling. In addition, epithelial injury biomarkers, such as NGAL, which is produced not only by neutrophils but also by RTECs, show a significant increase in AAV and correlate with disease activity. This highlights the dual roles of immune cells and tubular epithelium in causing renal injury. Markers of tubular injury, such as kidney injury molecule-1 (KIM-1), have been shown to be strongly associated with interstitial inflammation and disease activity. This provides strong evidence that damage to tubular epithelial cells is a major characteristic of disease pathogenesis rather than a simple secondary effect [81,82,83,84,85].

At the cellular level, when RTECs are continually exposed to inflammatory mediators and oxidative stress, they undergo apoptosis, lose epithelial integrity, and acquire a profibrotic phenotype. These cells begin to produce ECM proteins and secrete fibrogenic mediators, such as TGF-β and gremlin, both of which have been localized in tubular epithelial cells and correlate with disease severity in crescentic ANCA-associated glomerulonephritis. In addition, annexin A1 levels in renal tissue are proportional to the extent of renal injury and linked to neutrophil-driven inflammation, indicating a mutual relationship between immune and epithelial cells [79,81,86].

### 4.5. Kidney Transplantation

Ischemia–reperfusion injury (IRI) is an unavoidable consequence of kidney transplantation and a major contributor to delayed graft function and long-term graft survival. The proximal tubule cells in the kidney are most vulnerable to reduced blood supply because of their high energy requirements and reliance on oxidative phosphorylation. During ischemia, oxygen deprivation leads to ATP depletion, mitochondrial dysfunction, and accumulation of metabolic intermediates. When reperfusion occurs, the sudden influx of oxygen leads to ROS production, calcium overload, and an inflammatory response, resulting in cell death via apoptosis or necrosis of RTECs. Through TLR signaling, hypoxia-inducible factor signaling, and the sphingolipid signaling cascade, RTECs become a potent source of proinflammatory mediators [87,88,89,90]. RTECs amplify the inflammatory response by producing cytokines, chemokines, and DAMPs, thereby recruiting leukocytes and promoting innate immune activation. This self-reinforcing loop of epithelial injury and inflammation is central to the development of acute tubular injury and subsequent graft dysfunction [88,90,91,92].

Beyond their role in IRI, RTECs directly contribute to alloimmune activation during kidney transplantation. Although traditionally considered non-professional cells, RTECs can express MHC class II molecules and co-stimulatory signals under inflammatory conditions, enabling antigen presentation. Experimental evidence shows that human proximal tubular epithelial cells express HLA-DR, CD80, and ICAM-1 and can activate CD4^+^ T cells, thereby contributing to direct allorecognition [92,93,94]. This ability makes RTECs substantial contributors to the mediation of T cell-driven rejection. During acute cellular rejection, inflammatory infiltrates rich in T cells and macrophages accumulate in the interstitium and spread to tubular structures, a process called tubulitis, which is a characteristic feature of rejection pathology. In addition, RTECs facilitate this event by releasing chemokines and cytokines that attract and ‘keep’ T cells in the tubular epithelium [92,94,95]. In addition, inflammatory cytokines such as interferon can drive RTECs to increase the expression of antigen-presenting components, thereby amplifying local immune responses and leading to ongoing damage to the transplanted organ [92,93].

In the context of T cell-dependent rejection, epithelial damage is not only a consequence of direct immune-mediated cell killing but also results from inflammatory signals that drive extensive epithelial dedifferentiation and dysfunction of the entire nephron. This change makes RTECs more vulnerable to immune cell invasion while reducing their regenerative capacity. Acute injury is linked to chronic graft dysfunction through this mechanism [94,95,96]. One of the main characteristics of chronic allograft injury is the development of tubulointerstitial fibrosis and tubular atrophy (TA/IF), which are the final steps leading to graft failure. The key event in this progression is the phenotypic change in RTECs to a profibrotic state [97,98].

In kidney transplant injuries, tubular epithelial cells can undergo partial EMT. This change is characterized by a reduction in epithelial markers and the acquisition of mesenchymal traits, including vimentin expression and collagen production. It remains unclear whether RTECs fully transform into fibroblasts during EMT. However, cells that undergo only partial EMT release profibrotic cytokines and extracellular matrix (ECM) components, leading to interstitial fibrosis [98,99,100]. The induction of EMT in renal epithelial cells is a key event in the pathogenesis of kidney fibrosis. It relies on activation of the canonical TGF-β/Smad pathway as well as other pathways, including Wnt/β-catenin, all of which coordinate the transcriptional reprogramming of epithelial cells and fibrogenesis. In particular, EMT-associated alterations can be induced post-transplantation and in stable grafts immediately, and they largely predict the onset of chronic allograft nephropathy [100,101,102]. EMT-related changes in kidney grafts can be detected early after transplantation, even in clinically stable grafts, and are highly predictive of the later development of chronic allograft nephropathy. Kidney transplants often fail because of early tubular damage. That damage manifests as the production of proteins such as NGAL and KIM-1 by tubular cells. These markers correlate with late graft dysfunction, acute injuries, and overall organ survival, and they also serve as indicators of health during the recovery phase [97,98,103]. In transplant tissue tests, KIM-1 is expressed only in proximal tubule cells that are injured. Its level correlates with how poorly the kidney functions. This makes it a sensitive signal for early injury. The results show that tubular injury is not just a side effect; it is key to whether a transplant succeeds or fails [104,105,106].

### 4.6. Toxic and Drug-Induced Nephropathy

Toxic and drug-induced nephropathy (TIN) drives many cases of AKI and is a top reason for CKD. Patients exposed to drugs such as chemotherapy, antibiotics, calcineurin inhibitors, or radiocontrast media face the highest risk. Although these substances differ greatly, damage usually centers on RTECs, mostly those in the proximal tubule. These cells are affected first because they rely heavily on oxidative energy production, and actively move foreign compounds using transporters such as organic cation and anion transporters. Their intense workload leaves them particularly prone to stress and harm [107,108]. When exposed to nephrotoxic agents like cisplatin, RTECs experience severe cellular stress that is well marked by mitochondrial dysfunction, ATP depletion, and the generation of ROS. ROS disrupt cellular homeostasis, leading to the formation of peroxidized lipids, DNA damage, and activation of stress-responsive signaling pathways, including p53 and MAPK cascades. Eventually, cells may die by either apoptosis or necrosis, depending on the intensity and duration of the injury. KIM-1 has been identified as a highly specific marker of proximal tubular damage, expressed exclusively in injured tubular epithelial cells and strongly correlated with renal dysfunction [107,109].

RTECs are not only responsible for directly producing cytotoxic damage but also for driving enhanced inflammatory responses. Upon injury, tubular epithelial cells release pro-inflammatory cytokines (such as TNF-α and IL-6), chemokines (such as MCP-1/CCL2), and DAMPs. These molecules activate Toll-like receptors (TLRs) and other pattern recognition receptors. As a result, immune cells such as macrophages and neutrophils are recruited, initiating a continuous inflammatory cycle that not only causes further epithelial injury but also leads to tissue damage. This epithelial-immune cell crosstalk is a hallmark of toxic nephropathy and explains why an acute injury may develop into a chronic condition [110,111,112]. Simultaneously, RTECs initiate multiple cellular stress response pathways, including autophagy and ER stress signaling. Initially, autophagy serves as a defense mechanism by degrading dysfunctional mitochondria and proteins, thereby maintaining cellular integrity. Yet, if the stress is excessive, these cellular adaptation mechanisms may become ineffective, leading to maladaptive repair and tubular dysfunction. For example, autophagy has opposing roles in kidney injury and immunerelated renal disease, where this process regulates both epithelial cell survival and inflammatory signaling [83,113].

An essential step in the progression of toxic nephropathy is the conversion of acute injury into chronic fibrosis. Phenotypic changes in RTECs are responsible for this conversion. After prolonged stress, RTECs undergo a partial EMT, lose epithelial characteristics, and acquire mesenchymal features, including increased vimentin expression and the release of ECM components. Still, unlike cells undergoing full EMT, RTECs remain within the tubular basement membrane and adopt a profibrotic secretory phenotype, producing key fibrotic factors such as TGF-β and collagen. These cells do not fully differentiate into fibroblasts, but they are crucial in driving tubulointerstitial fibrosis through paracrine signaling and ECM deposition. This idea aligns with the current understanding of epithelial plasticity, which recognizes pEMT as a principal driver of renal fibrosis [99,100,114].

### 4.7. Genetic and Metabolic Disorders Affecting RTECs

#### 4.7.1. Metabolic Disorders

RTECs are key targets in the pathogenesis of metabolic kidney injury. In diabetic kidney disease (DKD), chronic hyperglycemia, disrupted lipid metabolism, and excessive mitochondrial ROS production each trigger tubular stress. Abnormal glycolipid metabolism induces oxidative stress, mitochondrial injury, lipid accumulation, cell death, and inflammation in RTECs, positioning them as key players in the initiation and progression of DKD [115]. Diabetic RTEC injury forms an interconnected network: hyperglycemia initiates ROS and advanced glycation end-product (AGE) accumulation, which drives inflammatory and apoptotic cascades that, in turn, stimulate transforming growth factor (TGF)-β-dependent and mechanically induced fibrogenesis. This reciprocal amplification explains why tubular injury is a strong predictor of DKD progression and systemic complications such as cardiovascular disease [116].

#### 4.7.2. Hereditary Disorders

Cystinosis and hereditary Fanconi syndromes highlight the unique vulnerability of PTECs to lysosomal, metabolic, and trafficking defects. *CTNS* mutations cause cystine accumulation, oxidative stress, impaired autophagy, abnormal vesicular trafficking, and energy dysregulation, collectively resulting in severe proximal tubular dysfunction and renal Fanconi syndrome, the earliest and most prominent manifestation of cystinosis [117]. Cystinosis impairs V-ATPase subunit expression and disrupts intracellular pH, lysosomal clearance, and mitochondrial energetics in human and CTNS^−/−^ RTECs. Restoring ATP6V0A1 or administering antioxidant treatment rescues mitochondrial function and autophagic flux, supporting the central role of tubular epithelial metabolic dysfunction in disease progression [118]. RTEC injury is the dominant phenotype, leading to progressive renal failure and eventual need for kidney transplantation [119].

In patients with Alport syndrome, the STING pathway in kidney tubule cells is hyperactivated, resulting in phosphorylation of TANK-binding kinase 1 (TBK1). This is only one step in the chain of events whereby a signal is transmitted, leading to the activation of other transcription factors, such as NF-kB and interferon regulatory factor 3 (IRF3). This pathway leads to the generation and secretion of proinflammatory cytokines and chemokines, including CCL2, CCL5, CXCL10, TNF-α, and IL-1, which are major drivers of the inflammatory response and of macrophage attraction and retention in the renal interstitium. Genetically altered or stressed tubular epithelial cells have a stronger reaction to inflammation-causing agents because they produce more cytokines. Drug-mediated blockade of the STING pathway leads to a significant reduction in cytokine expression, immune cell infiltration, and tubulointerstitial damage, highlighting the major role of tubular epithelial cell-intrinsic innate immune activation in the progression of kidney disease [120].

Mutations in the *OCRL1* gene result in dysfunction of tubular epithelial cells by disrupting phosphoinositide metabolism and endocytic trafficking. Proximal tubular cells in the kidney of patients with Lowe syndrome and Dent-2 disease depend heavily on OCRL1-regulated phosphoinositide turnover for two major functions: maintaining endocytic capacity and apical membrane organization. If the OCRL1 function is missing, phosphatidylinositol(4,5)-bisphosphate (PtdIns(4,5)P) accumulates in the endosome membranes. This results in unregulated remodeling of the actin cytoskeleton, altered endosomal trafficking, and defective receptor recycling at the apical surface of tubular epithelial cells [121]. Mainly due to impaired endosomal trafficking and acidification, receptor-mediated endocytosis is less efficient in proximal tubular epithelial cells in Dent’s disease. The dysfunction of key endocytic receptors, such as megalin and cubilin, is a direct consequence. The capture of filtered proteins is reduced, leading to the main symptom of the disease, namely, low molecular weight proteinuria [122]. When *OCRL1* is knocked out or mutated in RTECs, it causes excessive ROS generation, phosphatidylserine exposure on the cell surface, and activation of apoptotic cascades, leading to tubular cell injury and necrosis. In addition to inducing apoptosis, ROS also alter the physicochemical characteristics of membranes, thereby facilitating crystalline binding to tubular epithelial cells. This may cause nephrocalcinosis and tubular damage. The evidence obtained ties genetic abnormalities in Dent’s disease to oxidative stress-mediated damage of RTECs [123].

In ADTKD-UMOD, genetic alterations in the *UMOD* gene cause the uromodulin protein to misfold and become trapped within RTECs, primarily in the distal tubules. This leads to endoplasmic reticulum (ER) stress and activation of the unfolded protein response, which increases levels of the transcription factor CHOP. Continuous activation of CHOP causes EMT, production of the ECM, and the development of tubulointerstitial fibrosis, thus creating an exact molecular connection between protein accumulation inside cells and fibrosis driven by tubular epithelial cells [124].

## 5. Diagnostic Potential of RTECs

**Figure 4:**
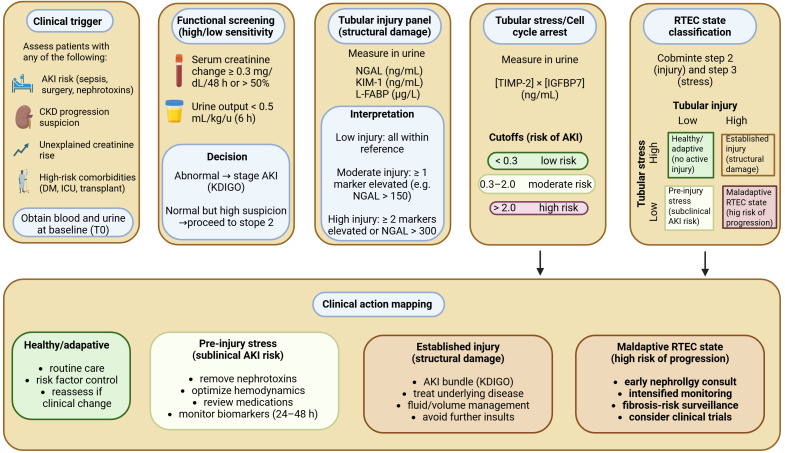
Proposed RTEC-centered diagnostic workflow integrating functional, injury, and stress biomarkers to guide clinical decision-making. It represents a step-by-step strategy for recognizing patients who face the risk of acute kidney injury (AKI) or whose state of kidney disease is deteriorating. It combines time-tested clinical signals with the latest renal tubular epithelial cell (RTEC)-derived biomarkers. If one suspects that the patient has risk factors for AKI (e. g., sepsis, surgery, nephrotoxins), that there is a suspicion of progression of chronic kidney disease (CKD), an unexplained rise in creatinine, or the patient has extremely severe conditions, then the process initiating after a visit in the doctor’s office, blood and urine samples (T0) will be taken. Step 1: Functional screening involves checking for changes in serum creatinine (>0.3 mg/dL within 48 h or >50%) and a decrease in urine output (<0.5 mL/kg/h for 6 h), thereby enabling staging of AKI per KDIGO criteria. If the functional parameters are normal but there remains a strong clinical suspicion, additional tests ought to be conducted. Step 2: Evaluation of tubular injury can be one of the means of determining the extent of injury. Occupationally exposed individuals who have sustained an injury may exhibit a rapid increase in very early urinary biomarkers, including neutrophil gelatinase-associated lipocalin (NGAL), kidney injury molecule-1 (KIM-1), and liver-type fatty acid-binding protein (L-FABP). The level of injury is classified as low, moderate, or high, based on the number and concentration of the abnormal markers. Step 3: Tubular stress and cell-cycle arrest are assessed using the urinary biomarker duo [tissue inhibitor of matrix metalloproteinase-2 (TIMP-2)] × [insulin-like growth factor binding protein 7 (IGFBP7)], which enables risk stratification for AKI (low, moderate, or high risk). Step 4: RTEC state classification integrates injury (Step 2) and stress (Step 3) signals to define four biologically informed states: (i) healthy/adaptive (no injury), (ii) pre-injury stress (subclinical AKI risk), (iii) established injury (structural damage), and (iv) maladaptive RTEC state (high risk of progression). These situations range from simple monitoring and risk-factor control to quite radical interventions, such as removing nephrotoxins, hemodynamic optimization, implementation of AKI care bundles, and intensified monitoring or early referral to nephrologists for high-risk patients, in addition to routine monitoring and risk-factor control. Essentially, this structure makes a case for integrating functional markers with tubular injury and stress biomarkers, which might help with risk stratification and even individual management decisions, though further validation and standardization would be necessary before routine clinical implementation.

RTECs form a highly specialized, injury-sensitive interface within the nephron. Their unique metabolic burden, polarized structure, and transporter-rich phenotype make them primary sensors and reporters of renal stress. Consequently, biomarkers, EVs, multi-omics, and functional imaging modalities derived from RTECs may become key diagnostic tools (Figure 4). They may aid early detection and offer mechanistic insights, but translation to routine precision medicine is not yet established.

### 5.1. Blood and Urine Biomarkers Derived from RTECs

RTEC injury triggers rapid molecular changes detectable in urine and blood, enabling early detection of epithelial stress before overt functional decline is reflected in serum creatinine levels. Kidney injury markers such as neutrophil gelatinase-associated lipocalin (NGAL), kidney injury molecule-1 (KIM-1), liver-type fatty acid-binding protein (L-FABP), and the cell-cycle arrest biomarkers tissue inhibitor of matrix metalloproteinase-2 (TIMP-2)/insulin-like growth factor binding protein 7 (IGFBP-7) provide the earliest indications of structural tubular damage (Table 2). Systematic analyses show that NGAL, KIM-1, and L-FABP predict AKI development earlier than functional biomarkers, with NGAL and KIM-1 demonstrating high diagnostic accuracy across diverse AKI settings. Prospective studies confirm that L-FABP and NGAL rise substantially in community-acquired AKI, whereas TIMP-2/IGFBP-7 remains the best-performing marker for early tubular stress and risk stratification [12,13].

Alongside damage indicators, stress and dedifferentiation markers, such as cysteine-rich angiogenic inducer 61 (CYR61), PENK, and other injury-response molecules, reflect epithelial maladaptation characterized by ER stress, cytoskeletal remodeling, apoptosis, and inflammatory activation. These markers discriminate among etiologies of tubular injury and correlate with severity and prognosis [5]. Tubular transport dysfunction, a hallmark of proximal tubular impairment, is reflected by biomarkers such as β_2_-microglobulin, which rises when reabsorptive capacity is compromised. Controlled studies in healthy kidney donors showed increases in β_2_-microglobulin and NGAL after nephrectomy, confirming their sensitivity for detecting subtle proximal tubular transport defects [125].

### 5.2. Extracellular Vesicles and RTEC-Derived Exosomes

Various types of EVs, such as exosomes and microvesicles, have become highly sensitive, accurate, and cell-type-specific indicators of RTEC biology. EVs are lipid bilayer-enclosed nanoparticles secreted by almost all renal epithelial cell types, including proximal and distal tubule cells, collecting duct cells, and podocytes. Their molecular contents, including proteins, lipids, mRNAs, microRNAs, and characteristic tetraspanins, precisely mirror the cells’ physiological conditions, the stress environment, and injury-related signaling pathways from which they are derived. Urinary EVs (uEVs) have become a popular non-invasive method for assessing kidney health because they are easy to collect and carry tubule-segment-specific characteristics [125]. The release of EVs from RTECs is an active, regulated process that is substantially amplified under injury conditions, including hypoxia, oxidative stress, inflammation, and mechanical strain. Under these conditions, RTEC-derived EVs exhibit marked alterations in abundance and composition, including enrichment of stress-response proteins, injury-associated signaling molecules, and transporters that undergo dysregulation during tubular injury. These vesicles thus function as dynamic “liquid biopsies” of tubular health, capturing early molecular perturbations long before functional decline becomes clinically evident [126].

The diagnostic application of urinary EVs has advanced rapidly. In CKD, uEVs show distinct changes in concentration, size distribution, and proteomic content that correlate with disease severity, estimated glomerular filtration rate (eGFR), and albuminuria. Profiling the uEV proteome has revealed disease-specific changes, including alterations in transporter levels, cytoskeletal remodeling proteins, and fibrosis-associated molecules. Notably, recent quantitative proteomics in pediatric CKD identified uEV signatures capable of detecting early nephron loss and congenital hypoplasia. A newly developed ELISA-based platform successfully validated these signatures in a clinical context, demonstrating their translational potential for early diagnosis [2,127].

While RTEC-derived EVs show cell-type specificity and potential for non-invasive monitoring, significant challenges remain regarding standardization, validation, and clinical translation. Their therapeutic potential is mainly experimental and unproven in humans [40].

### 5.3. Multi-Omics Approaches

Multi-omics approaches have expanded our understanding of RTEC injury, but lack validated thresholds and remain resource-intensive. Their utility for guiding clinical decisions is not yet established. Transcriptomic profiling using bulk RNA-seq, single-cell RNA sequencing (scRNA-seq), ATAC-seq, and spatial transcriptomics has identified variations in tubular cell states in both healthy and diseased kidneys. Extensive multimodal human kidney maps have shown that epithelial cells in anatomically defined nephron segments also exhibit significant regional differences in transcriptional identity, metabolic programs, and chromatin accessibility. For example, simultaneous scRNA-seq and single-cell ATAC-seq profiling of over 440,000 human kidney cells has shown that PTECs in diseased tissue undergo injury-induced dedifferentiation, metabolic rewiring, and lipid-handling abnormalities, with transcriptional and epigenomic signatures varying sharply by anatomical location. Such multimodal datasets have also revealed that RTEC injury involves activation of stress pathways, inflammatory transcriptional programs, and unique epigenetic configurations not captured by bulk measurements. Furthermore, single-cell epigenetic profiling provides a mechanistic framework for understanding how changes in chromatin accessibility shape maladaptive RTEC behaviors, enabling the dissection of gene-regulatory mechanisms underlying heterogeneous injury states [33,128].

Proteomics and metabolomics add complementary layers of biological information, enabling detection of post-translational modifications, oxidative and metabolic stress markers, and protein–protein interaction networks that are invisible to transcriptomics. Proteome-wide analyses in CKD and AKI have identified injury-specific protein signatures, alterations in cytoskeletal components, and remodeling of metabolic and signaling pathways tightly linked to RTEC dysfunction and fibrosis. Spatial metabolomics studies have uncovered pathogenic metabolite accumulation, most notably adenine deposition in fibrotic regions of DKD, and have demonstrated that regional metabolite patterns functionally correlate with transcriptomic and proteomic injury signatures. This integrated metabolomic view provides essential functional context for understanding RTEC stress biology and supports the development of metabolite-targeted diagnostics and therapeutics [36,129].

Epigenomic signatures provide a durable molecular record of tubular injury. Epigenetic remodeling, including changes in enhancer activation, transcription-factor binding landscapes, and chromatin accessibility, occurs early in the AKI-to-CKD transition. Single-cell multi-omics approaches that profile chromatin accessibility and transcription at single-cell resolution now enable direct linking of epigenomic states to transcriptional outcomes, clarifying the regulatory logic that drives maladaptive epithelial repair and persistent dedifferentiation. Through such integrative analyses, injury-primed RTEC populations can be distinguished from adaptively repairing ones, offering novel biomarkers and targets for intervention [33,128].

## 6. Conclusions

RTECs are now recognized as key players in the development of kidney disease and in natural healing processes. Single-cell and multi-omics studies have uncovered distinct RTEC injury states. Omics-based approaches are revealing features of cellular injury and interactions that were previously inaccessible, yet translating these results into certified clinical instruments remains an ongoing challenge. Large, multicenter, longitudinal studies in humans are needed to address issues such as small patient cohorts, poor model translation, and a lack of testing standards. These studies would also use consistent sample collection and laboratory analysis methods. Shared biobanks with urine, blood, and kidney tissues linked to detailed medical records will help demonstrate that results can be replicated across different research efforts. Integration of cutting-edge technologies should move beyond descriptive analyses toward actionable study designs. In particular, single-cell RNA sequencing (scRNA-seq), single-cell ATAC-seq, spatial transcriptomics, and proteomics should be applied in parallel within the same patient samples to build multi-layered atlases of RTEC injury and repair. Such integrative approaches will enable the identification of causal regulatory networks rather than isolated biomarkers. In addition, longitudinal multi-omics profiling of uEVs is a practical, minimally invasive strategy for monitoring dynamic RTEC states. Coupling uEV profiling with machine learning algorithms and clinical metadata can support the development of predictive models for disease progression, treatment response, and early detection of subclinical injury.

Real-world testing fails without simple, practical biomarkers. Future research must translate complex omics data into clear, practical tests, such as multiplex assays or focused protein patterns, that fit into routine clinics. This shift makes lab findings usable outside the lab. Lab-grown kidney tissues and human-derived organ models show how real organs respond. These tools offer a smarter alternative to animal testing for evaluating drug pathways and treatments in people’s own cell types. They also validate biological activity where it matters most. Kidney multi-omics research needs clear rules for how data is integrated, reported, and validated. These shared practices will accelerate precision nephrology by enabling fair comparisons across studies and helping new biomarkers gain regulatory approval.

Focusing on RTECs could mark a major shift in nephrology, moving biomarker research toward precision medicine. Deep sequencing of individual kidney cells and their environment would yield new models that capture variations in kidney tubule disease states, rather than merely measuring injury markers. Future diagnostic platforms could be based on methods for profiling uEVs, other circulating biomarkers, and high-dimensional omics signatures, combined with machine learning algorithms to produce real-time “tubular health scores”. Such compound metrics could facilitate the earliest detection of subclinical injury, the distinction between adaptive and maladaptive repair states, and the prediction of the individual patient’s disease course.

## Figures and Tables

**Table 1:** RTEC stress responses and consequences.

Stress Mechanism	Key Features	Molecular Drivers	Outcome
Mitochondrial dysfunction	FAO ↓ OXPHOS ↓ ATP depletion	AMPK ↓ PGC-1α ↓ CPT1 ↓ OXPHOS ↓	Energy failure ROS accumulation Tubular atrophy and fibrosis
Oxidative stress	Reactive oxygen species accumulation Lipid, protein, and DNA oxidation Activation of inflammatory signaling	NADPH oxidase ↑ NF-κB activation Nrf2 imbalance	Inflammation (IL-6, TNF-α) Endothelial dysfunction Cell injury and death
UPR/ER stress	Protein misfolding in the endoplasmic reticulum Unfolded protein response activation Translation attenuation	PERK IRE1 ATF6 CHOP ↑	Apoptosis (CHOP-mediated) Senescence induction Reduced regenerative capacity
Cell-cycle arrest	G1/S or G2/M arrest DNA damage response activation p53/p21 pathway activation	p53 ↑ p21 ↑ ATM/ATR Cyclins ↓	Impaired proliferation Maladaptive repair AKI to CKD transition
Senescence and SASP	Irreversible cell-cycle arrest Senescence-associated secretory phenotype Secretion of cytokines and chemokines	p16^INK4a^ ↑ p21^CIP1^ ↑ IL-6 ↑ CXCL8 ↑ CCL2 ↑	Chronic inflammation Fibroblast activation fibrosis CKD progression
Metabolic reprogramming (AKI, sepsis)	Shift toward glycolysis Impaired lipid metabolism Accumulation of lipid droplets	GLUT1 ↑ HK2 ↑ PFKFB3 ↑ FAO ↓ PPARα ↓ mTOR ↑	Short-term survival advantage Long-term atrophy and fibrosis Vulnerability to recurrent injury
Metabolic reprogramming (CKD)	Sustained glycolytic metabolism Reduced mitochondrial biogenesis Epigenetic remodeling	HIF-1α ↑ LDHA ↑ PDK4 ↑ DNMTs ↑ HDACs ↑	Tubular dedifferentiation ECM deposition Progressive CKD fibrosis

Abbreviations: AKI: Acute kidney injury; AMPK: AMP-activated protein kinase; ATF6: Activating Transcription Factor 6; ATM: Ataxia-Telangiectasia Mutated; ATR: Ataxia-Telangiectasia and Rad3-related; CCL2: C-C Motif Chemokine Ligand 2; CHOP: C/EBP Homologous Protein; CKD: Chronic kidney disease; CPT1: Carnitine Palmitoyltransferase 1; CXCL8: C-X-C Motif Chemokine Ligand 8; DNA: Deoxyribonucleic acid; DNMTs: DNA Methyltransferases; ECM: Extracellular Matrix; ER: Endoplasmic reticulum; FAO: Fatty Acid Oxidation; GLUT1: Glucose Transporter 1; G1/S: Gap 1 to synthesis phase transition of the cell cycle; G2/M: Gap 2 to mitosis phase transition of the cell cycle; HDACs: Histone Deacetylases; HIF-1α: Hypoxia-Inducible Factor 1-alpha; HK2: Hexokinase 2; IL-6: Interleukin-6; IRE1: Inositol-Requiring Enzyme 1; LDHA: Lactate Dehydrogenase A; mTOR: Mechanistic Target of Rapamycin; NF-κB: Nuclear Factor kappa-light-chain-enhancer of activated B cells; Nrf2: Nuclear Factor Erythroid 2-related Factor 2; OXPHOS: Oxidative Phosphorylation; PDK4: Pyruvate Dehydrogenase Kinase 4; PERK: PKR-like ER Kinase; PFKFB3: 6-Phosphofructo-2-Kinase/Fructose-2,6-Bisphosphatase-3; PGC-1α: Peroxisome-Proliferator-Activated Receptor Gamma Co-activator 1-alpha; p16INK4a: Cyclin-Dependent Kinase Inhibitor 2A; p21CIP1: Cyclin-Dependent Kinase Inhibitor 1A; PPARα: Peroxisome Proliferator-Activated Receptor alpha; ROS: Reactive Oxygen Species; RTECs: Renal Tubular Epithelial Cells; SASP: Senescence-associated secretory phenotype; TNF-α: tumor necrosis factor-alpha; UPR: Unfolded protein response; ↑ Increase; ↓ decrease.

**Table 2:** RTEC-derived biomarkers in kidney disease.

Category	Biomarker	Type	What It Reflects
Injury	NGAL	Protein	Early tubular damage
Injury	KIM-1	Protein	Proximal tubular damage
Injury	L-FABP	Protein	Oxidative stress
Stress	TIMP-2/IGFBP-7	Cell-cycle marker	Protein
Dysfunction	β2-microglobulin	Protein	Reabsorption defect
Dedifferentiation	CYR61	Protein	Stress response
Dedifferentiation	PENK	Peptide	Injury signaling
Dedifferentiation	uEV cargo	Multi-omics	Cell-specific injury

Abbreviations: β2-microglobulin: Beta-2-microglobulin; CYR61: Cysteine-rich Angiogenic Inducer 61; IGFBP-7: Insulin-Like Growth Factor Binding Protein-7; KIM-1: Kidney Injury Molecule-1; L-FABP: Liver-type Fatty Acid-Binding Protein; NGAL: Neutrophil Gelatinase-Associated Lipocalin; PENK: Proenkephalin; TIMP-2: Tissue Inhibitor of Metalloproteinases-2; uEV: urinary Extracellular Vesicle.

## Data Availability

No new data were created or analyzed in this study.

## Abbreviations

The following abbreviations are used in this manuscript:

AGE	Advanced Glycation End-product
AKI	Acute Kidney Injury
AngII	Angiotensin II
AP-1	Activator Protein-1 (transcription factor complex)
ATAC-seq	Assay for Transposase-Accessible Chromatin using sequencing
ATF6	Activating Transcription Factor 6
ATP	Adenosine Triphosphate
ATR	Angiotensin receptor
CCL2	C-C Motif Chemokine Ligand 2 (Monocyte Chemoattractant Protein-1)
CCR2	C-C Motif Chemokine Receptor 2
CFTR	Cystic fibrosis transmembrane conductance regulator
CKD	Chronic Kidney Disease
CSF1R	Colony-stimulating factor 1 receptor
CTNS	Cystinosin (gene mutated in cystinosis)
CXCL10	C-X-C Motif Chemokine Ligand 10
CXCL16	C-X-C Motif Chemokine Ligand 16
CXCR3	C-X-C Chemokine Receptor 3
CXCR6	C-X-C Chemokine Receptor 6
CYR61	Cysteine-rich protein 61 (injury/stress-induced marker)
DAMP	Damage-associated molecular pattern
DCT	Distal Convoluted Tubule
DKD	Diabetic Kidney Disease
DNA	Deoxyribonucleic Acid
ECM	Extracellular Matrix
ELISA	Enzyme-Linked Immunosorbent Assay
EMT	Epithelial-to-Mesenchymal Transition
ER	Endoplasmic Reticulum
EV	Extracellular Vesicle
L-FABP	Liver-type Fatty Acid-Binding Protein
GFR	Glomerular Filtration Rate
GWAS	Genome-Wide Association Study
HAVCR1 (KIM-1)	Hepatitis A Virus Cellular Receptor 1/Kidney Injury Molecule-1
IGFBP-7	Insulin-like Growth Factor Binding Protein-7
iIFTA	Inflammation in interstitial fibrosis and tubular atrophy
IL	Interleukin
IRE1	Inositol-Requiring Enzyme 1
KIM-1	Kidney Injury Molecule-1
KPMP	Kidney Precision Medicine Project
MAPK	Mitogen-activated protein kinase
ML	Machine Learning
MR	Mineralocorticoid receptor
NCC	Sodium Chloride Cotransporter
NF-κB	Nuclear Factor kappa-light-chain-enhancer of activated B cells
NFAT5	Nuclear Factor of Activated T Cells 5
NGAL	Neutrophil Gelatinase-Associated Lipocalin
NK	Natural Killer (immune cell)
NKCC2	Na^+^-K^+^-2Cl^−^ Cotransporter 2
NLRP3	NLR Family Pyrin Domain-Containing 3 (inflammasome component)
NNMT	Nicotinamide-N-Methyltransferase
PDGF	Platelet-Derived Growth Factor
PDGFR	Platelet-Derived Growth Factor Receptor
PENK	Proenkephalin (tubular stress marker)
PERK	PKR-like ER Kinase
PT	Proximal Tubule
RNA	Ribonucleic Acid
ROS	Reactive Oxygen Species
RTEC	Renal Tubular Epithelial Cell
SASP	Senescence-Associated Secretory Phenotype
STAT	Signal transducer and activator of transcription
STING	Stimulator of Interferon Genes
TA/IF	Tubular atrophy/interstitial fibrosis
TAL	Thick Ascending Limb
TAZ	Transcriptional Co-Activator with PDZ-binding Motif
TGF-β	Transforming Growth Factor-β
TIMP-2	Tissue Inhibitor of Metalloproteinases-2
TMEM16A	Transmembrane protein 16A (or anoctamin-1)
TNF	Tumor Necrosis Factor
TRPV5	Transient Receptor Potential Cation Channel Subfamily V Member 5
UPR	Unfolded Protein Response
VCAM1	Vascular Cell Adhesion Molecule-1
YAP	Yes-Associated Protein
